# (*E*)-*N*′-(4-Hydroxy­benzyl­idene)-2-methoxy­benzohydrazide

**DOI:** 10.1107/S1600536808029334

**Published:** 2008-09-24

**Authors:** Xue-Hui Zhan

**Affiliations:** a College of Chemistry and Biological Engineering, Changsha University of Science and Technology, Changsha 410076, People’s Republic of China

## Abstract

The title compound, C_15_H_14_N_2_O_3_, exists in the *E* configuration with respect to the central methyl­idene unit. The dihedral angle between the two substituted benzene rings is 22.0 (2)°. Within the mol­ecule there is an intra­molecular N—H⋯O hydrogen bond involving the hydro­zide H atom and the O atom of the meth­oxy substituent on the adjacent phenyl ring. In the crystal structure, mol­ecules are linked through inter­molecular O—H⋯O hydrogen bonds, forming zigzag chains along the *b* direction.

## Related literature

For bond-length data, see: Allen *et al.* (1987 ▶). For background on the biological properties of hydrazones, see: El-Tabl *et al.* (2008 ▶); Chen *et al.* (2008 ▶); Alvarez *et al.* (2008 ▶); Ventura & Martins (2008 ▶); Kalinowski *et al.* (2008 ▶). For related structures, see: Diao & Yu (2006 ▶); Shan *et al.* (2008 ▶); Fun *et al.* (2008 ▶); Yehye *et al.* (2008 ▶); Ejsmont *et al.* (2008 ▶); Han *et al.* (2006 ▶); Lu *et al.* (2008 ▶).
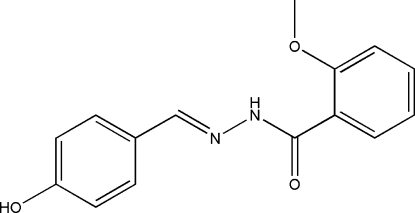


## Experimental

### 

#### Crystal data

C_15_H_14_N_2_O_3_
*M* *_r_* = 270.28
Orthorhombic, 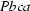

*a* = 13.113 (3) Å
*b* = 9.189 (2) Å
*c* = 22.110 (4) Å
*V* = 2664.2 (10) Å^3^
*Z* = 8
Mo *K*α radiation
μ = 0.10 mm^−1^
*T* = 298 (2) K
0.10 × 0.10 × 0.08 mm

#### Data collection

Bruker SMART 1000 CCD area-detector diffractometer
Absorption correction: multi-scan (*SADABS*; Bruker, 2001 ▶) *T*_min_ = 0.991, *T*_max_ = 0.992
20705 measured reflections
2899 independent reflections
1656 reflections with *I* > 2σ(*I*)
*R*_int_ = 0.084

#### Refinement

*R*[*F* ^2^ > 2σ(*F* ^2^)] = 0.055
*wR*(*F* ^2^) = 0.136
*S* = 1.02
2899 reflections
186 parameters
1 restraint
H atoms treated by a mixture of independent and constrained refinement
Δρ_max_ = 0.15 e Å^−3^
Δρ_min_ = −0.16 e Å^−3^

### 

Data collection: *SMART* (Bruker, 2007 ▶); cell refinement: *SAINT* (Bruker, 2007 ▶); data reduction: *SAINT*; program(s) used to solve structure: *SHELXTL* (Sheldrick, 2008 ▶); program(s) used to refine structure: *SHELXTL*; molecular graphics: *SHELXTL*; software used to prepare material for publication: *SHELXTL*.

## Supplementary Material

Crystal structure: contains datablocks global, I. DOI: 10.1107/S1600536808029334/su2061sup1.cif

Structure factors: contains datablocks I. DOI: 10.1107/S1600536808029334/su2061Isup2.hkl

Additional supplementary materials:  crystallographic information; 3D view; checkCIF report

## Figures and Tables

**Table 1:** Hydrogen-bond geometry (Å, °)

*D*—H⋯*A*	*D*—H	H⋯*A*	*D*⋯*A*	*D*—H⋯*A*
N2—H2*A*⋯O3	0.895 (10)	1.91 (2)	2.620 (2)	135 (2)
O1—H1⋯O2^i^	0.82	1.90	2.700 (2)	164

Symmetry code: (i) 
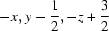
.

**Fig. 1.**
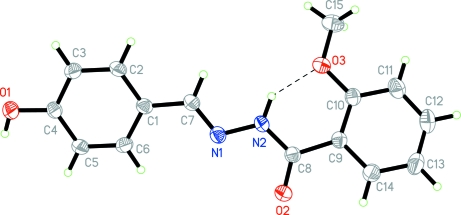
The molecular structure of compound (I), with 30% probability displacement ellipsoids for non-H atoms.

**Fig. 2.**
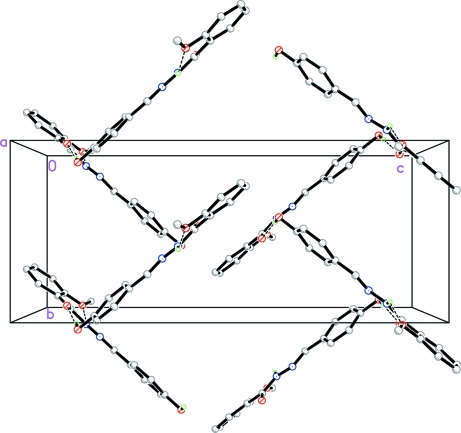
The crystal packing of compound (I), viewed along the a axis. Hydrogen bonds are shown as dashed lines.

## supplementary crystallographic information

### Comment

Hydrazones derived from the reactions of aldehydes with hydrazides show potential biological properties (El-Tabl *et al.*, 2008; Chen *et al.*, 2008; Alvarez *et al.*, 2008; Ventura & Martins, 2008; Kalinowski *et al.*, 2008). In the last few years, a large number of hydrazones have been reported (Diao & Yu, 2006; Shan *et al.*, 2008; Fun *et al.*, 2008; Yehye *et al.*, 2008; Ejsmont *et al.*, 2008). As a continuous study, the crystal structure of the title compound, (I), is reported in this paper.

Compound (I) was prepared by the reaction of 4-hydroxybenzaldehyde and 2-methoxybenzohydrazide in methanol. The molecular structure of compound (I) is illustrated in Fig. 1. The C7—N1 bond length of 1.272 (3) Å indicates the presence of a typical C═N double bond. The molecule exists in the E configuration with respect to the methylidene unit (C7═N1), as observed in similar compounds (Han *et al.*, 2006; Lu *et al.*, 2008). The dihedral angle between the two substituted benzene rings is 22.02 (12)°, indicating that the molecule is not planar. In the 2-methoxyphenyl unit atom C15 deviates slightly from the mean plane of the benzene ring (C9–C14) by 0.097 (3) Å. The bond lengths are in normal ranges (Allen *et al.*, 1987).

In the molecule there is an intramolecular N—H···O hydrogen bond (Table 1), and in the crystal structure symmetry related molecules are linked through intermolecular O—H···O hydrogen bonds (Table 1), forming zig-zag chains along the b direction (Fig. 2).

### Experimental

2-Methoxybenzohydrazide (0.166 g, 1 mmol) was dissolved in ethanol (50 ml), then 4-hydroxybenzaldehyde (0.122 g, 1 mmol) was added slowly to the solution, and the mixture was heated at reflux with continuous stirring for 1 h. The solution was cooled to room temperature, yielding colorless crystallites. Recrystallization from absolute ethanol yielded block-like single crytals of compound (I).

### Refinement

The NH H-atom (H2A) was located in a difference Fourier map and freely refined, with the N—H distance restrained to 0.90 (1) Å, and *U*_iso_ = 0.08 Å^2^. The remaining OH and C-bound H-atoms were included in calculated positions and treated as riding atoms: O—H = 0.82 Å with *U*_iso_(H) = 1.5*U*_eq_(O), and C—H = 0.93–0.96 Å, with *U*_iso_(H) = 1.2 or 1.5*U*_eq_(C).

### Crystal data

**Table:**

C_15_H_14_N_2_O_3_	*F*(000) = 1136
*M**_r_* = 270.28	*D*_x_ = 1.348 Mg m^−^^3^
Orthorhombic, *P**b**c**a*	Mo *K*α radiation, λ = 0.71073 Å
Hall symbol: -P 2ac 2ab	Cell parameters from 1422 reflections
*a* = 13.113 (3) Å	θ = 2.4–24.5°
*b* = 9.189 (2) Å	µ = 0.10 mm^−^^1^
*c* = 22.110 (4) Å	*T* = 298 K
*V* = 2664.2 (10) Å^3^	Block, colourless
*Z* = 8	0.10 × 0.10 × 0.08 mm

### Data collection

**Table:**

Bruker SMART 1000 CCD area-detector diffractometer	2899 independent reflections
Radiation source: fine-focus sealed tube	1656 reflections with *I* > 2σ(*I*)
graphite	*R*_int_ = 0.084
ω scans	θ_max_ = 27.0°, θ_min_ = 1.8°
Absorption correction: multi-scan (SADABS; Bruker, 2001)	*h* = −16→16
*T*_min_ = 0.991, *T*_max_ = 0.992	*k* = −11→11
20705 measured reflections	*l* = −28→28

### Refinement

**Table:**

Refinement on *F*^2^	Primary atom site location: structure-invariant direct methods
Least-squares matrix: full	Secondary atom site location: difference Fourier map
*R*[*F*^2^ > 2σ(*F*^2^)] = 0.055	Hydrogen site location: inferred from neighbouring sites
*wR*(*F*^2^) = 0.136	H atoms treated by a mixture of independent and constrained refinement
*S* = 1.02	*w* = 1/[σ^2^(*F*_o_^2^) + (0.0456*P*)^2^ + 0.6248*P*] where *P* = (*F*_o_^2^ + 2*F*_c_^2^)/3
2899 reflections	(Δ/σ)_max_ < 0.001
186 parameters	Δρ_max_ = 0.15 e Å^−^^3^
1 restraint	Δρ_min_ = −0.16 e Å^−^^3^

### Special details

**Table:**

Geometry. All e.s.d.'s (except the e.s.d. in the dihedral angle between two l.s. planes) are estimated using the full covariance matrix. The cell e.s.d.'s are taken into account individually in the estimation of e.s.d.'s in distances, angles and torsion angles; correlations between e.s.d.'s in cell parameters are only used when they are defined by crystal symmetry. An approximate (isotropic) treatment of cell e.s.d.'s is used for estimating e.s.d.'s involving l.s. planes.
Refinement. Refinement of *F*^2^ against ALL reflections. The weighted *R*-factor *wR* and goodness of fit *S* are based on *F*^2^, conventional *R*-factors *R* are based on *F*, with *F* set to zero for negative *F*^2^. The threshold expression of *F*^2^ > σ(*F*^2^) is used only for calculating *R*-factors(gt) *etc*. and is not relevant to the choice of reflections for refinement. *R*-factors based on *F*^2^ are statistically about twice as large as those based on *F*, and *R*- factors based on ALL data will be even larger.

### Fractional atomic coordinates and isotropic or equivalent isotropic displacement parameters (Å^2^)

**Table:**

	*x*	*y*	*z*	*U*_iso_*/*U*_eq_	
O1	0.05041 (12)	0.41453 (19)	0.87757 (7)	0.0566 (5)	
H1	−0.0077	0.4422	0.8850	0.085*	
O2	0.12254 (12)	1.04261 (19)	0.58301 (7)	0.0603 (5)	
O3	0.43319 (11)	1.01975 (19)	0.60152 (7)	0.0565 (5)	
N1	0.18804 (13)	0.8432 (2)	0.66327 (8)	0.0445 (5)	
N2	0.25132 (14)	0.9141 (2)	0.62338 (8)	0.0460 (5)	
C1	0.18233 (16)	0.6684 (2)	0.74406 (10)	0.0423 (6)	
C2	0.23796 (17)	0.5671 (3)	0.77706 (10)	0.0488 (6)	
H2	0.3071	0.5555	0.7690	0.059*	
C3	0.19308 (17)	0.4837 (3)	0.82142 (10)	0.0493 (6)	
H3	0.2319	0.4168	0.8430	0.059*	
C4	0.09138 (16)	0.4992 (2)	0.83370 (9)	0.0413 (6)	
C5	0.03475 (17)	0.5996 (3)	0.80141 (11)	0.0537 (7)	
H5	−0.0343	0.6112	0.8097	0.064*	
C6	0.07971 (17)	0.6823 (3)	0.75726 (10)	0.0527 (7)	
H6	0.0405	0.7489	0.7358	0.063*	
C7	0.23330 (17)	0.7529 (3)	0.69767 (10)	0.0467 (6)	
H7	0.3032	0.7404	0.6928	0.056*	
C8	0.21358 (17)	1.0145 (2)	0.58522 (9)	0.0415 (5)	
C9	0.28755 (16)	1.0927 (2)	0.54622 (9)	0.0409 (5)	
C10	0.39297 (17)	1.1018 (3)	0.55572 (10)	0.0440 (6)	
C11	0.4525 (2)	1.1908 (3)	0.51948 (11)	0.0585 (7)	
H11	0.5224	1.1971	0.5263	0.070*	
C12	0.4087 (2)	1.2695 (3)	0.47368 (12)	0.0667 (8)	
H12	0.4489	1.3305	0.4501	0.080*	
C13	0.3060 (2)	1.2592 (3)	0.46224 (12)	0.0642 (7)	
H13	0.2769	1.3110	0.4305	0.077*	
C14	0.24661 (19)	1.1715 (3)	0.49838 (10)	0.0532 (6)	
H14	0.1771	1.1647	0.4905	0.064*	
C15	0.53806 (18)	1.0379 (3)	0.61787 (12)	0.0671 (8)	
H15A	0.5805	1.0137	0.5840	0.101*	
H15B	0.5540	0.9750	0.6512	0.101*	
H15C	0.5499	1.1372	0.6294	0.101*	
H2A	0.3188 (8)	0.900 (3)	0.6214 (12)	0.080*	

### Atomic displacement parameters (Å^2^)

**Table:**

	*U*^11^	*U*^22^	*U*^33^	*U*^12^	*U*^13^	*U*^23^
O1	0.0478 (10)	0.0620 (11)	0.0600 (10)	0.0031 (9)	0.0133 (8)	0.0151 (9)
O2	0.0392 (10)	0.0760 (13)	0.0656 (11)	0.0092 (9)	0.0079 (8)	0.0192 (10)
O3	0.0400 (9)	0.0684 (12)	0.0611 (10)	−0.0044 (8)	−0.0049 (8)	0.0097 (9)
N1	0.0417 (11)	0.0500 (12)	0.0419 (10)	−0.0011 (9)	0.0048 (9)	0.0008 (10)
N2	0.0366 (10)	0.0537 (13)	0.0477 (11)	0.0013 (10)	0.0096 (9)	0.0093 (10)
C1	0.0372 (12)	0.0498 (14)	0.0400 (12)	0.0021 (11)	0.0026 (10)	−0.0010 (11)
C2	0.0329 (12)	0.0626 (17)	0.0509 (14)	0.0050 (11)	0.0069 (10)	0.0057 (13)
C3	0.0444 (14)	0.0528 (16)	0.0508 (14)	0.0087 (12)	0.0029 (11)	0.0081 (12)
C4	0.0412 (13)	0.0441 (14)	0.0386 (12)	−0.0025 (11)	0.0044 (10)	−0.0017 (11)
C5	0.0340 (12)	0.0695 (18)	0.0575 (15)	0.0055 (12)	0.0067 (11)	0.0122 (13)
C6	0.0430 (14)	0.0646 (18)	0.0506 (14)	0.0104 (12)	0.0004 (11)	0.0132 (13)
C7	0.0394 (13)	0.0546 (16)	0.0460 (13)	0.0040 (12)	0.0080 (10)	0.0026 (12)
C8	0.0407 (13)	0.0460 (14)	0.0377 (12)	0.0023 (11)	0.0025 (10)	−0.0014 (11)
C9	0.0436 (13)	0.0385 (13)	0.0407 (12)	0.0015 (11)	0.0049 (10)	−0.0015 (11)
C10	0.0482 (14)	0.0440 (14)	0.0399 (12)	−0.0019 (11)	0.0030 (10)	−0.0025 (11)
C11	0.0566 (16)	0.0604 (18)	0.0584 (16)	−0.0162 (13)	0.0065 (13)	−0.0018 (14)
C12	0.083 (2)	0.0601 (18)	0.0573 (16)	−0.0179 (16)	0.0138 (15)	0.0089 (15)
C13	0.082 (2)	0.0573 (18)	0.0537 (15)	0.0071 (16)	0.0087 (14)	0.0144 (14)
C14	0.0518 (14)	0.0564 (17)	0.0514 (14)	0.0062 (13)	0.0036 (12)	0.0038 (13)
C15	0.0461 (15)	0.076 (2)	0.0793 (19)	−0.0062 (14)	−0.0128 (14)	−0.0078 (16)

### Geometric parameters (Å, °)

**Table:**

O1—C4	1.355 (2)	C5—H5	0.9300
O1—H1	0.8200	C6—H6	0.9300
O2—C8	1.222 (3)	C7—H7	0.9300
O3—C10	1.368 (3)	C8—C9	1.484 (3)
O3—C15	1.432 (3)	C9—C14	1.389 (3)
N1—C7	1.272 (3)	C9—C10	1.401 (3)
N1—N2	1.375 (2)	C10—C11	1.386 (3)
N2—C8	1.344 (3)	C11—C12	1.371 (4)
N2—H2A	0.895 (10)	C11—H11	0.9300
C1—C6	1.383 (3)	C12—C13	1.373 (4)
C1—C2	1.389 (3)	C12—H12	0.9300
C1—C7	1.450 (3)	C13—C14	1.377 (3)
C2—C3	1.377 (3)	C13—H13	0.9300
C2—H2	0.9300	C14—H14	0.9300
C3—C4	1.368 (3)	C15—H15A	0.9600
C3—H3	0.9300	C15—H15B	0.9600
C4—C5	1.383 (3)	C15—H15C	0.9600
C5—C6	1.370 (3)		
			
C4—O1—H1	109.5	O2—C8—N2	122.0 (2)
C10—O3—C15	119.53 (19)	O2—C8—C9	120.8 (2)
C7—N1—N2	114.27 (18)	N2—C8—C9	117.2 (2)
C8—N2—N1	120.36 (19)	C14—C9—C10	117.7 (2)
C8—N2—H2A	115.6 (18)	C14—C9—C8	116.2 (2)
N1—N2—H2A	124.1 (18)	C10—C9—C8	125.9 (2)
C6—C1—C2	117.5 (2)	O3—C10—C11	122.4 (2)
C6—C1—C7	123.3 (2)	O3—C10—C9	117.3 (2)
C2—C1—C7	119.2 (2)	C11—C10—C9	120.3 (2)
C3—C2—C1	121.5 (2)	C12—C11—C10	120.1 (3)
C3—C2—H2	119.2	C12—C11—H11	119.9
C1—C2—H2	119.2	C10—C11—H11	119.9
C4—C3—C2	120.0 (2)	C11—C12—C13	120.8 (3)
C4—C3—H3	120.0	C11—C12—H12	119.6
C2—C3—H3	120.0	C13—C12—H12	119.6
O1—C4—C3	118.0 (2)	C12—C13—C14	119.2 (3)
O1—C4—C5	122.7 (2)	C12—C13—H13	120.4
C3—C4—C5	119.4 (2)	C14—C13—H13	120.4
C6—C5—C4	120.4 (2)	C13—C14—C9	121.9 (2)
C6—C5—H5	119.8	C13—C14—H14	119.0
C4—C5—H5	119.8	C9—C14—H14	119.0
C5—C6—C1	121.2 (2)	O3—C15—H15A	109.5
C5—C6—H6	119.4	O3—C15—H15B	109.5
C1—C6—H6	119.4	H15A—C15—H15B	109.5
N1—C7—C1	123.9 (2)	O3—C15—H15C	109.5
N1—C7—H7	118.1	H15A—C15—H15C	109.5
C1—C7—H7	118.1	H15B—C15—H15C	109.5

### Hydrogen-bond geometry (Å, °)

**Table:**

*D*—H···*A*	*D*—H	H···*A*	*D*···*A*	*D*—H···*A*
N2—H2A···O3	0.90 (1)	1.91 (2)	2.620 (2)	135 (2)
O1—H1···O2^i^	0.82	1.90	2.700 (2)	164

Symmetry codes: (i) −*x*, *y*−1/2, −*z*+3/2.
